# Mn_3_O_4_/NiO Nanoparticles Decorated on Carbon Nanofibers as an Enzyme-Free Electrochemical Sensor for Glucose Detection

**DOI:** 10.3390/bios13020264

**Published:** 2023-02-13

**Authors:** Mengjie Li, Jie Dong, Dongmei Deng, Xun Ouyang, Xiaoxia Yan, Shima Liu, Liqiang Luo

**Affiliations:** 1 College of Sciences, Shanghai University, Shanghai 200444, China; 2 Key Laboratory of Hunan Forest Products and Chemical Industry Engineering, Jishou University, Jishou 416000, China

**Keywords:** electrospinning, Mn_3_O_4_/NiO nanoparticles, carbon nanofibers, glucose biosensor

## Abstract

Transition metal oxides have garnered a lot of attention in the field of electrocatalysis along with their unique crystal structure and excellent catalytic properties. In this study, carbon nanofibers (CNFs) decorated with Mn_3_O_4_/NiO nanoparticles were made using electrospinning and calcination. The conductive network constructed by CNFs not only facilitates electron transport, but also provides landing sites for nanoparticles, thus reducing nanoparticle aggregation and exposing more active sites. Additionally, the synergistic interaction between Mn_3_O_4_ and NiO improved electrocatalytic capacity for glucose oxidation. The Mn_3_O_4_/NiO/CNFs modified glassy carbon electrode shows satisfactory results in terms of linear range and anti-interference capability for glucose detection, suggesting that the constructed enzyme-free sensor has a promising application in clinical diagnosis.

## 1. Introduction

Electrochemical glucose sensors perform an indispensable role in monitoring glucose concentrations, which not only can be employed to control the intake of glucose in diet, but also to predict diseases associated with high or low glucose levels [1,2]. Enzyme-based sensors show excellent sensitivity and accuracy for glucose detection, but they are sensitive to temperature and pH which limits their storage and transportation [3]. In contrast, enzyme-free sensors have the advantage of being cheap, stable, and sensitive [4], thus attracting considerable attention in recent years.

Noble metals and their alloys have shown excellent catalytic properties and stability [5,6] and are often used as enzyme-free sensors for glucose detection [7,8]. However, the high cost and scarcity of resources limit wide applications of noble metals. In this regard, transition metal can overcome the drawbacks mentioned above for noble metals and be applied as catalysts for many reactions [9], such as CO_2_ utilization [10], oxygen evolution reaction [11], and oxygen reduction reaction [12,13]. Generally speaking, composite transition metal oxides exhibit higher catalytic capacity and lower catalytic potential than single transition metal oxide due to the synergistic effect between different metal oxides. Wang et al. synthesized CuCo_2_O_4_/NiO nanoneedle arrays, modified on carbon cloth by hydrothermal method, which were used as self-supporting electrodes for the glucose detection [14]. Xu et al. fabricated CuO-NiO nanocomposites for the detection of glucose which exhibited excellent selectivity, reproducibility, and long-time stability [15].

One-dimensional nanofibers possess low density, large aspect ratio, and high charge carrier directed mobility [16]. Thomas et al. grew α-MoO_3_ nanorods on glass substrates by thermal evaporation for ammonia sensing at room temperature [17]. Zhang et al. used a hydrothermal method with low temperature growth to synthesize Co_9_S_8_ nanotubes @CuS nanosheets, which were modified on glassy carbon electrode (GCE) for the detection of glucose and H_2_O_2_ [18]. Kokulnathan et al. synthesized TiO/Au nanofibers by electrospinning for the determination of diphenylamine in food [19]. Among the above methods, electrospinning is a simple, efficient, and inexpensive way to synthesize one-dimensional nanofibers [16,20]. Transition metal oxides/carbon nanofibers composites fabricated by electrospinning have the following advantages: (i) CNFs with large aspect ratio can form a conductive network, which facilitates the electron transport [21]; (ii) the mesh structure can serve as a substrate to support the nanoparticles which allows more active sites of the nanoparticles to be exposed; and, (iii) the synergistic interaction between transition metal oxides and carbon nanofibers can facilitate the electrocatalytic performance.

In this study, CNFs decorated with Mn_3_O_4_/NiO nanoparticles (Mn_3_O_4_/NiO/CNFs) were synthesized using electrospinning and subsequent heat treatment. The presence of CNFs not only increases the conductivity of the material, but also expands the active surface area of the catalysts. Additionally, the multivalence of transition metals facilitates the redox reaction at the electrode surface. The synergistic effect between Mn_3_O_4_ and NiO is beneficial to improve the catalytic performance of glucose oxidation. It is worth noting that the constructed sensor has a good range of linearity, low detection limit, and outstanding interference immunity, indicating that the constructed enzyme-free sensor is promising in clinical diagnosis.

## 2. Experimental Section

### 2.1. Materials

Polyacrylonitrile (PAN) was supplied by Sigma-Aldrich (St. Louis, MO, USA). Mn(Ac)_2_∙4H_2_O, N,N-dimethylformamide (DMF), NaOH, glycocoll (Gly), Ni(Ac)_2_∙4H_2_O, and glucose (Glu) were supported by Sinopharm Chemical Reagent Co., Ltd. (Shanghai, China). Sucrose (Suc), dopamine (DA), maltose (Mal), ascorbic acid (AA), fructose (Fru), uric acid (UA), and NaCl were bought from Alfa Aesar Co., Ltd. (Shanghai, China). Millipore water (18.25 MΩ cm) was used.

### 2.2. Instrumentations

The scanning electron microscope (SEM, SU5000, HITACHI, Tokyo, Japan) was utilized to observe the morphology and size of materials. The high-resolution transmission electron microscope (HRTEM; JEM 2100F, JEOL, Tokyo, Japan) was further used to investigate the microscopic crystal structure of samples. X-ray diffraction (XRD, D/MAX2200, Rigaku, Tokyo, Japan) was employed to characterize the crystal structure of the materials. Fourier transform infrared spectroscopy (FTIR, AVATAR370, Thermo Scientific, Waltham, MA, USA) was performed to study chemical structure. The degree of graphitization of CNFs was studied by Raman spectrum (INVIA, Renishaw, London, UK). X-ray photoelectron spectrometer (XPS, ESCALAB-250XI, Thermo Scientific, Waltham, MA, USA) was applied to measure the surface components of materials in qualitative detail.

### 2.3. Synthesis of Mn_3_O_4_/NiO/CNFs

**Scheme 1:**
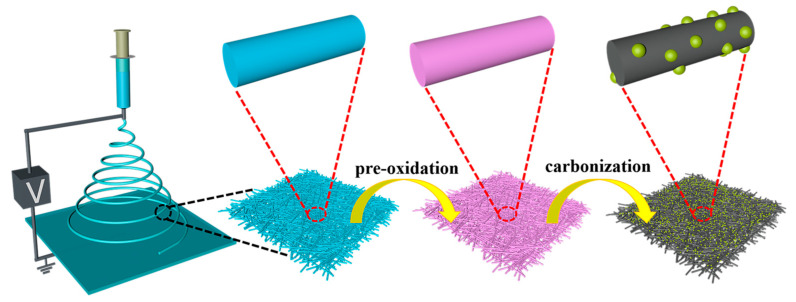
Schematic diagram of synthesis procedure of Mn_3_O_4_/NiO/CNFs.

The synthesis process of Mn_3_O_4_/NiO/CNFs is illustrated in Scheme 1. Firstly, 0.5 g of PAN was mixed with 10 mL DMF and agitated to form a transparent solution. Then, 0.068 g Mn(Ac)_2_·4H_2_O and 0.017 g Ni(Ac)_2_·4H_2_O were added to the above solution and stirred continuously. Afterwards, the obtained brown precursor solution was moved to a 10 mL plastic injection syringe, and delivered to the metal needle at a constant stream rate of 0.6 mL h^−1^ under control of an injection pump. The grounded aluminum plate was used as the collector and kept away from the needle at a distance of 12 cm. After electrospinning, the collected precursor fibers were dried overnight at 60 °C under vacuum. The dried precursor fibers were pre-oxidized in air at 250 °C for 2 h, and then carbonized at 800 °C for another 2 h in an Ar stream. For comparison, Mn_3_O_4_/CNFs and NiO/CNFs were synthesized by the same method.

### 2.4. Electrochemical Measurements

The electrochemical measurements were carried out in a three-electrode system consisting of a platinum wire as counter electrode, Ag/AgCl (saturated KCl) as reference electrode, and Mn_3_O_4_/NiO/CNFs/GCE as working electrode. The three-electrode system was connected to an electrochemical workstation (CHI 842D, CH Instruments, Shanghai, China). The Mn_3_O_4_/NiO/CNFs/GCE was prepared in following steps: the bare GCE was polished with aluminum powder (0.5 μm and 0.01 μm), followed by cleaning with deionized water. Then, 5 μL of Mn_3_O_4_/NiO/CNFs uniform dispersion was dropped on GCE and then dried under infrared light to obtain Mn_3_O_4_/NiO/CNFs/GCE for glucose detection. The supporting electrolyte was 0.1 M NaOH.

## 3. Results and Discussion

### 3.1. XRD, Raman, FT-IR, and XPS Characterization of Mn_3_O_4_/NiO/CNFs

**Figure 1:**
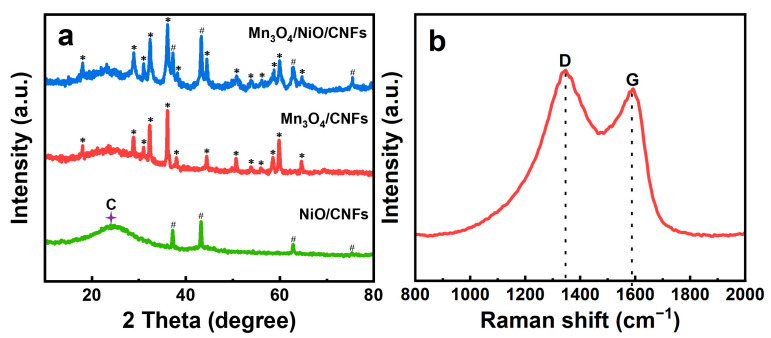
(**a**) XRD patterns of Mn_3_O_4_/CNFs, NiO/CNFs, and Mn_3_O_4_/NiO/CNFs; (**b**) Raman spectrum of Mn_3_O_4_/NiO/CNFs at 800 °C.

The crystal structure and purity of Mn_3_O_4_/NiO/CNFs were characterized with XRD. Figure 1a shows the XRD patterns of Mn_3_O_4_/CNF, NiO/CNF, and Mn_3_O_4_/NiO/CNF. The obtained material contained a set of diffraction peaks that matched with Mn_3_O_4_, NiO, and CNF. In addition, there were no other impure phases. The peak at 24.2° could be ascribed to the (200) plane of CNF. The diffraction peaks at 37.2°, 43.2°, 62.8°, and 75.4° displayed the crystal planes of (111), (200), (220), and (311) plane for NiO [22]. The diffraction peaks at 17.9°, 28.9°, 31.0°, 32.3°, 36.1°, 38.0°, 44.4°, 50.7°, 53.1°, 56.0°, 58.5°, 59.9°, and 64.5° were well indexed to the (101), (112), (200), (103), (211), (004), (220), (105), (312), (303), (321), (224), and (400) plane of Mn_3_O_4_ with a cubic spinel [23].

The Raman spectrum was applied to evaluate the degree of graphitization of CNFs. In Figure 1b, the Raman spectrum shows a couple of significant peaks at 1350 and 1590 cm^−1^ which were directly related to the D band and G band, respectively [24]. The intensity of the D band reflected the carbon defects, while the intensity of the G band gave information about the graphitic structure. The intensity ratio between the D and G band, R = I_D_/I_G_, can be used to reflect the degree of graphitization of CNFs [25]. The R value is 1.07, which means that there are still defects in the material under calcination at 800 °C. The explanation for the defect is that the N element in PAN has not been completely removed. As shown in Figure S1, an FT-IR spectrum of Mn_3_O_4_/NiO/CNFs was performed. The band at 3444 cm^−1^ is identified as the stretching vibration of O-H, which is related to the adsorption of water. The peak at 1620 cm^−1^ can correspond to the C=C bond. The weak peak observed at 1383 cm^−1^ can be attributed to the C-H bond. The band at about 1169 cm^−1^ is caused by C-O [26]. The peaks which appear at 618 cm^−1^ and 523 cm^−1^ can be traced back to Mn_3_O_4_ and NiO, respectively [27].

**Figure 2:**
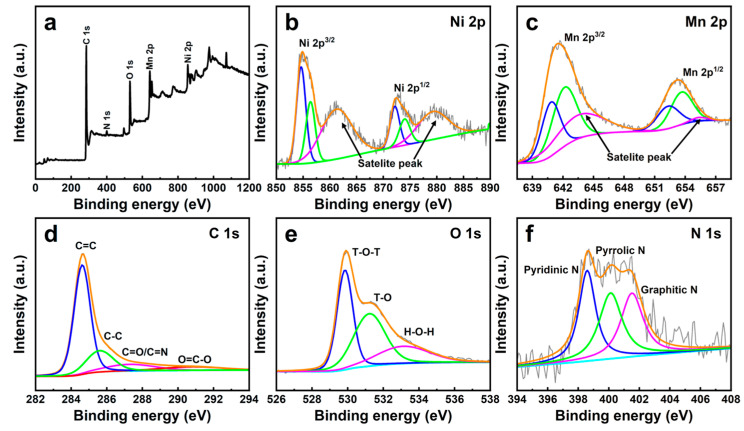
XPS spectra of Mn_3_O_4_/NiO/CNFs: (**a**) full wide-scan; (**b**) Ni 2p; (**c**) Mn 2p; (**d**) C 1s; (**e**) O 1s; and (**f**) N 1s.

The elemental composition and valence state of the Mn_3_O_4_/NiO/CNFs were reflected using XPS technique. The fully scanned spectrum (Figure 2a) demonstrates that the synthesized materials contained elements of C, N, O, Ni, and Mn. The peaks located at 854.7 eV and 872.1 eV were associated with Ni 2p_3/2_ and Ni 2p_1/2_, which were derived from Ni^2+^ (Figure 2b). In addition, two satellite peaks accompanying the main peaks were observed at 861.5 eV and 879.8 eV, proving the presence of Ni^2+^ from NiO [28]. The peaks located at 856.5 eV and 874.0 eV in Ni 2p spectrum corresponded to Ni 2p_3/2_ and Ni 2p_1/2_ from Ni^3+^, respectively. Here, more energy was required to form Ni^3+^ than Ni^2+^, since the compensation of large amounts of charge leads to the introduction of Ni vacancies and oxygen gaps, which in turn cause the formation of Ni^3+^ [29,30]. As shown in Figure 2c, the Mn 2p spectrum consists of a pair of major peaks located at 653.3 eV and 641.6 eV. The deconvolution peaks at the binding energies of 640.9 eV/652.4 eV and 642.3 eV/653.7 eV were caused by Mn^2+^ and Mn^3+^ species, respectively. The peaks located at about 645.0 eV and 655.0 eV belong to satellite peaks. Four peaks with energies of 284.6 eV, 285.6 eV, 287.4 eV, and 290.1 eV, corresponding to C=C, C-C, C=O/C=N, and O=C-O bonds, respectively, were fitted from the high-resolution C 1s peak (Figure 2d) [31,32]. The O 1s spectrum can be divided into three characteristic peaks, where the peaks of T-O-T and T-OH (T symbolizes transition metal) were observed at 529.9 eV and 531.2 eV, respectively. Moreover, the binding energy value of O 1s located at 533.2 eV represents the water-adsorbed on the surface (H-O-H) (Figure 2e). In the spectrogram of N 1s (Figure 2f), three peaks located at 398.8 eV, 400.1 eV, and 402.0 eV can be considered as pyridinic N, pyrrolic N, and graphitic N, respectively. Among them, pyridinic N and pyrrolic N are the two main doping forms in CNF [33].

### 3.2. SEM and TEM of Mn_3_O_4_/NiO/CNFs

**Figure 3:**
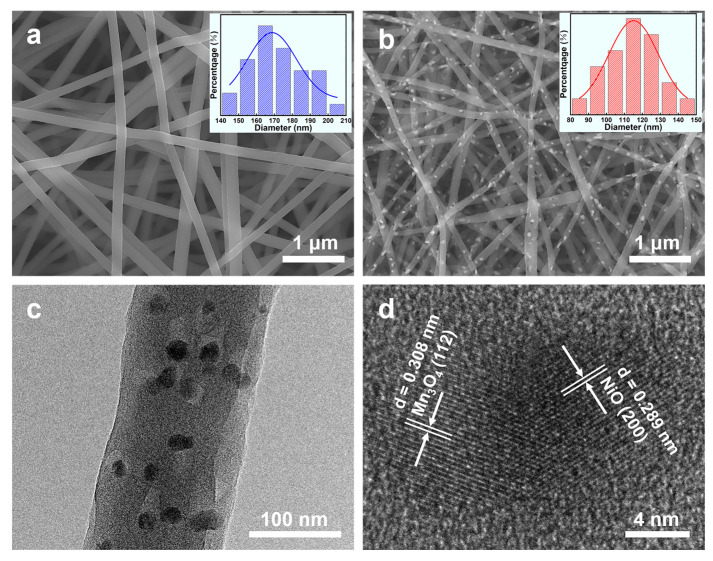
SEM images of electrospun: (**a**) Mn(Ac)_2_/Ni(Ac)_2_/PAN nanofibers and (**b**) Mn_3_O_4_/NiO/CNFs; (**c**) TEM image of Mn_3_O_4_/NiO/CNFs; (**d**) HRTEM image of Mn_3_O_4_/NiO.

The surface morphologies of Mn(Ac)_2_/Ni(Ac)_2_/PAN precursor nanofibers and Mn_3_O_4_/NiO/CNFs were observed through SEM. In Figure 3a, the surface of uncalcined precursor nanofibers was smooth due to the amorphous structure of Ni(Ac)_2_ and Mn(Ac)_2_. After calcination at 800 °C, the nanoparticles were uniformly dispersed on the nanofiber surface (Figure 3b). The previous XRD result confirmed that the nanoparticle consisted of Mn_3_O_4_ and NiO. The diameter statistics and Gaussian distribution of the precursor nanofibers and Mn_3_O_4_/NiO/CNFs are shown in the insets of Figure 3a,b. Obviously, the average diameter of the nanofibers decreased from 168 to 115 nm after calcination, a phenomenon which was ascribed to the reduction of huge amount of H, O, and N elements during the carbonization process. TEM and HRTEM were carried out to further reveal the microscopic crystal structure of Mn_3_O_4_/NiO/CNF. As shown in Figure 3c, Mn_3_O_4_/NiO nanoparticles were uniformly adhered to the CNF surface. Lattice fringes with spacing of 0.308 nm and 0.289 nm corresponds to the (112) plane of Mn_3_O_4_ and (200) plane of NiO, respectively (Figure 3d) [34,35].

### 3.3. Electrochemical Characterization

**Figure 4:**
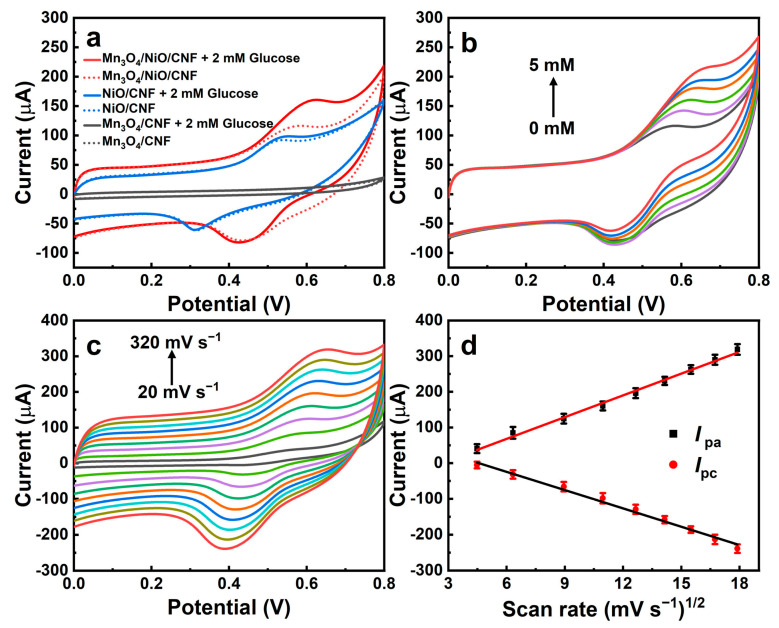
(**a**) CVs of Mn_3_O_4_/CNFs, NiO/CNFs, and Mn_3_O_4_/NiO/CNFs electrodes in the absence and presence of 2 mM glucose; (**b**) CVs of Mn_3_O_4_/NiO/CNFs electrode with different concentrations of glucose in 0.1 M NaOH at the scan rate of 100 mV s^−1^; (**c**) CVs of Mn_3_O_4_/NiO/CNFs electrode in the presence of 1 mM glucose at different scan rates; (**d**) peak currents in relationship to the square root of the scanning speed scan rate.

The cyclic voltammetry (CV) and electrochemical impedance spectroscopy (EIS) were used to study the electrochemical performance of Mn_3_O_4_/NiO/CNFs. EIS tests of different modified electrodes were conducted in the solution with 5 mM [Fe(CN)_6_]^3−/4−^ and 0.1 M KCl. The outcome of the EIS test is reflected by the Nyquist plot, where the semicircular part of the high frequency region relates to the electron transfer restriction process and the linear part of the low frequency region reflects the diffusion process. The decrease in the radius of the semicircle represents a decrease in the electron transfer resistance at the electrode interface (R_et_). In Figure S2, compared to Mn_3_O_4_/CNFs/GCE and NiO/CNFs/GCE, Mn_3_O_4_/NiO/CNFs/GCE shows the smallest diameter, indicating the fastest charge transfer capability. To investigate the electrochemical performance of Mn_3_O_4_/NiO/CNFs, CV was employed. Figure 4a shows the CV curves of Mn_3_O_4_/CNFs/GCE, NiO/CNFs/GCE, and Mn_3_O_4_/NiO/CNFs/GCE in 0.1 M NaOH solution with and without 2 mM glucose. The oxidation current that changed for Mn_3_O_4_/CNFs/GCE in the presence of glucose can be neglected. Meanwhile, NiO/CNFs/GCE exhibited a positive electrocatalytic oxidation peak current for glucose detection which can be ascribed to the transition between Ni^2+^ and Ni^3+^. Notably, Mn_3_O_4_/NiO/CNFs/GCE exhibited the highest oxidation current. The mechanism of glucose oxidation on Mn_3_O_4_/NiO/CNFs can be explained by the following equations [36]:

NiO + H_2_O → Ni(OH)_2_ (1)

Ni(OH)_2_ + OH^−^ → NiOOH + H_2_O + e^−^ (2)

NiOOH + C_6_H_12_O_6_ → Ni(OH)_2_ + C_6_H_10_O_6_ (Gluconolactone) (3)

Originally, in the alkaline medium, NiO strongly adsorbed water molecules to form Ni(OH)_2_ (Equation (1)). Next, Ni(OH)_2_ was oxidized to NiOOH as electron mediator (Equation (2)). Then, NiOOH obtained electrons from glucose to form Ni(OH)_2_, and glucose was oxidized to gluconolactone (Equation (3)). Previous studies have indicated that Mn-based materials have a wide range for enzyme-free detection of glucose [37,38]. In this work, by virtue of the synergistic interaction between Mn and Ni, the composite exhibits more significant electrocatalytic activity for the oxidation of glucose [39]. The results of the EIS also confirm that the introduction of Mn_3_O_4_ can increase the electrical conductivity of the composites.

Figure 4b describes the CV curves of Mn_3_O_4_/NiO/CNFs/GCE in different glucose concentrations (from 0 mM to 5 mM). The oxidation current signal increased with increasing glucose concentration, indicating that Mn_3_O_4_/NiO/CNFs are suitable as electrocatalytic media for the construction of glucose sensors. To further investigate the kinetic process of glucose oxidation on the electrode surface, the CV curves of Mn_3_O_4_/NiO/CNFs/GCE were tested in the range of scan rate from 20 mV s^−1^ to 320 mV s^−1^ (Figure 4c). As illustrated in Figure 4d, the currents of the anodic peak (I_pa_) and cathodic peak (I_pc_) maintained a good linear connection with the square root of the scanning speed, indicating that the oxidation of glucose on Mn_3_O_4_/NiO/CNFs/GCE is a diffusion-controlled process.

**Figure 5:**
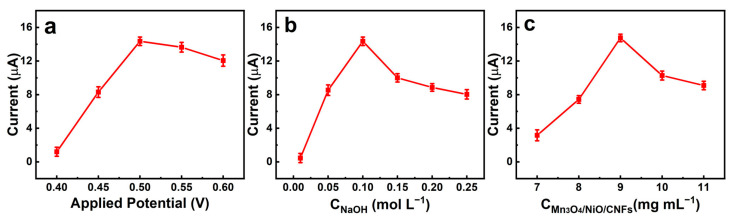
Optimization of parameters on the current response of 0.5 mM glucose: (**a**) applied potential; (**b**) the concentration of NaOH; (**c**) the content of Mn_3_O_4_/NiO/CNFs.

**Figure 6:**
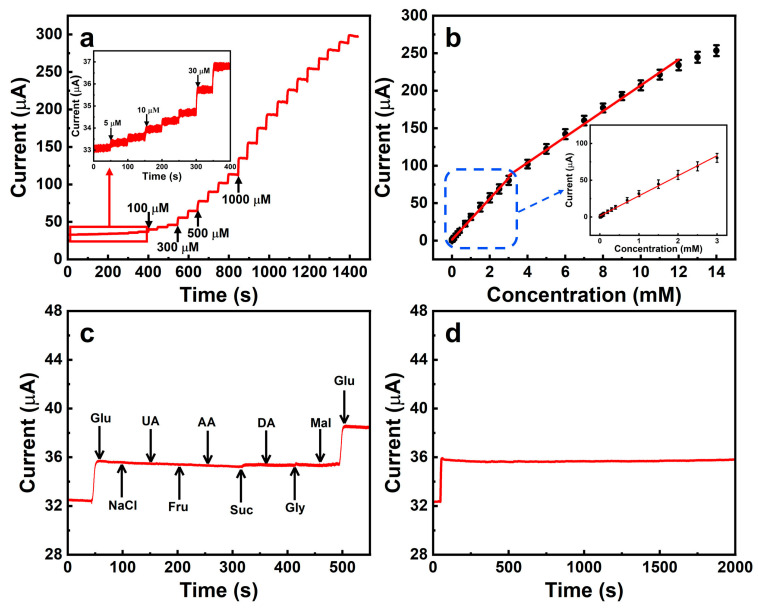
(**a**) Amperometric response of Mn_3_O_4_/NiO/CNFs electrode for different concentrations of glucose (inset is current response at low concentrations); (**b**) calibration curves corresponding to (**a**); (**c**) current response of Mn_3_O_4_/NiO/CNFs electrode to 100 μM glucose and different interferences; (**d**) the stability test of Mn_3_O_4_/NiO/CNFs electrode during the 2000 s. All the above experiments were conducted under optimal parameters.

To improve the electrocatalytic effect of Mn_3_O_4_/NiO/CNFs on glucose detection, the time-current method was used to optimize the parameters during the experiments, including the applied voltage and the concentrations of NaOH and Mn_3_O_4_/NiO/CNFs. Figure 5a shows that the response current increases with increasing applied voltage at the beginning, and then decreases when the applied voltage is higher than 0.50 V. Thus, 0.50 V is the optimum applied potential. In Figure 5b, the response current initially increases with increasing concentration of NaOH, and then starts to decrease when the concentration of NaOH exceeds 0.10 M. Therefore, 0.1 M NaOH was chosen as the optimum concentration of electrolyte. Figure 5c shows that the response current reaches its maximum when the concentration of Mn_3_O_4_/NiO/CNFs is 9 mg mL^−1^; therefore, 9 mg mL^−1^ was selected as the optimum modification material concentration. Figure 6a shows the current response of Mn_3_O_4_/NiO/CNFs/GCE for continuous addition of glucose under known optimal experimental conditions. The relationship between response current and glucose concentration shows linearly in the range of 5–3000 μM (R^2^ = 0.997) and 3000–12,000 μM (R^2^ = 0.993), and could be well-fitted to the equation: I (μA) = 27.33 C (mM) + 1.343 and I (μA) = 17.22 C (mM) + 35.015. The detection limit of Mn_3_O_4_/NiO/CNFs/GCE was calculated to be 0.73 μM (S/N = 3). The sensitivity was calculated to be 386.84 μA mM^−1^ cm^−2^ (5–3000 μM) and 243.74 μA mM^−1^ cm^−2^ (3000–12,000 μM). Additionally, the response time is a key factor in weighing up the practical application of the sensor. Figure S3 shows that the Mn_3_O_4_/NiO/CNFs/GCE at different glucose concentrations reaches its steady-state current within 2–4 s after the addition of glucose. Table 1 lists the electrocatalytic effects of some reported enzyme-free glucose sensors and enzymatic glucose sensors. Compared with them, our proposed Mn_3_O_4_/NiO/CNFs/GCE has a wider linear range as well as lower detection limit as glucose sensor.

### 3.4. Selectivity, Stability, Repeatability, and Reproducibility of Mn_3_O_4_/NiO/CNFs/GCE

The presence of other easily oxidized molecules in the actual sample may affect the accuracy of glucose detection. In Figure 6c, the addition of 5 μM of some interferences to 100 μM glucose solution at the ratio of 1:20 was investigated by a time-current test. The outcomes showed that the current responses caused by the addition of interferences were virtually negligible, indicating that Mn_3_O_4_/NiO/CNFs/GCE had good selectivity. Figure 6d shows the amperometric response of Mn_3_O_4_/NiO/CNFs/GCE to 100 μM glucose. The current kept stable during 2000 s, suggesting that the electrode had an excellent stability as glucose sensor. Besides, repeatability and reproducibility are also key to determine whether the synthesized materials can be used for actual sample detection. The five identical modified electrodes used to detect 0.1 mM glucose were found to have a relative standard deviation (RSD) of 6.38% (Figure S4), indicating that the electrodes have good reproducibility. The modified electrode was repeated five times to detect 0.1 mM glucose, and the RSD was calculated to be 1.32% (Figure S5), meaning that the electrode has a good repeatability. To evaluate the long-term stability of the sensor, the Mn_3_O_4_/NiO/CNFs/GCE was stored at room temperature for 30 days. The results show that the response current remains at 95.3% of the initial current after 30 days (Figure S6), indicating that our sensors have satisfactory long-term stability.

### 3.5. Actual Sample Analysis

To evaluate the practicality of Mn_3_O_4_/NiO/CNFs/GCE, serum samples with different concentrations of glucose were detected using standard addition method. The serum samples used in the experiment were from the School of Medicine, Shanghai University. The recoveries of the three samples were 100.09%, 100.20%, and 99.91%, respectively (Table 2), indicating that the built sensor can be accepted for glucose detection in serum samples.

## 4. Conclusions

In this work, Mn_3_O_4_/NiO/CNFs were synthesized by electrospinning and following calcination, and were used as an enzyme-free sensor for glucose detection. The intricate network structure of CNFs provides the backbone for the nanoparticles and facilitates electron transport. In addition, the synergistic effect between Mn_3_O_4_ and NiO is beneficial to improve the catalytic performance for glucose detection. The constructed Mn_3_O_4_/NiO/CNFs/GCE sensor has good linear range, low detection limit, and anti-interference ability, indicating that Mn_3_O_4_/NiO/CNFs have some application prospects as electrochemical sensors in clinical diagnosis.

## Figures and Tables

**Table 1:** Performance comparison of the proposed Mn_3_O_4_/NiO/CNFs/GCE with other glucose sensors.

Sensing Material	Linear Range (μM)	Detection Limit (μM)	Sensitivity (μA mM^−1^ cm^−2^)	Ref.
Mn_3_O_4_@ZIF-67/CC ^1^	0.8–6000	0.24	3421.0	[40]
CuCo_2_O_4_/NiO/CC	0.5–6000	0.28	4140	[14]
Cu_2_O-Cu-Au	0–4500	1.71	1082	[41]
Cu-Ni/NF	1–600	2	11,340.25	[42]
NiMoO_4_/CNF	0.3–4500	0.05	301.77	[43]
Ni_2_P-Cu_3_P	4–5000	0.1	4700	[44]
ZnO/MXene	50–700	17	29	[45]
Co_0_._33_Ni_0_._67_-HLDH ^2^	10–2000	3.1	242.9	[46]
Mn_3_O_4_/NiO	9.9–3665	1.0	226.2	[47]
MnO_2_/Co_3_O_4_	60–7000	0.03	127	[48]
FTO-CNTs/PEI/GOX	70–700	70	63.38	[49]
PEDOT: SCX/MXene/GOX/GCE	500–8000	22.5	-	[50]
Ni-MOF ^3^/rGO ^4^/CF ^5^	6–2090	0.6	852	[51]
Mn_3_O_4_/NiO/CNFs	5–3000 3000–12,000	0.73	386.84 243.74	This work

^1^ Carbon cloth. ^2^ Hollow layered double hydroxides. ^3^ Metal organic frameworks. ^4^ Reduced graphene oxide. ^5^ Carbon fiber.

**Table 2:** Analysis of glucose in human blood serum.

Sample	Detected (mM)	Commercial Method (mM)	RSD (%)	Recovery (%)
1	4.72	4.59	1.91	100.09
2	4.83	4.69	1.47	100.2
3	8.00	7.67	2.34	99.91

## Data Availability

Date will be made available on request.

## Supplementary Materials

The following supporting information can be downloaded at: https://www.mdpi.com/article/10.3390/bios13020264/s1, Figure S1: FT-IR spectrum of Mn_3_O_4_/NiO/CNFs.; Figure S2: EIS tests of the Mn_3_O_4_/CNFs/GCE, NiO/CNFs/GCE and Mn_3_O_4_/NiO/CNFs/GCE in 5.0 mM [Fe(CN)_6_]^3−/4−^ containing 0.1 M KCl.; Figure S3: Response time of (a) low glucose concentration and (b) high glucose concentration.; Figure S4: The reproducibility of the Mn_3_O_4_/NiO/CNFs/GCE.; Figure S5: The repeatability of the Mn_3_O_4_/NiO/CNFs/GCE.; Figure S6: The long-time stability.
