# Supplementary figures and images for: Mn3O4/NiO Nanoparticles Decorated on Carbon Nanofibers as an Enzyme-Free Electrochemical Sensor for Glucose Detection
Source: Biosensors (Basel). 2023 Feb 13;13(2):264. doi: 10.3390/bios13020264 (PMC9954078; doi:10.3390/bios13020264)

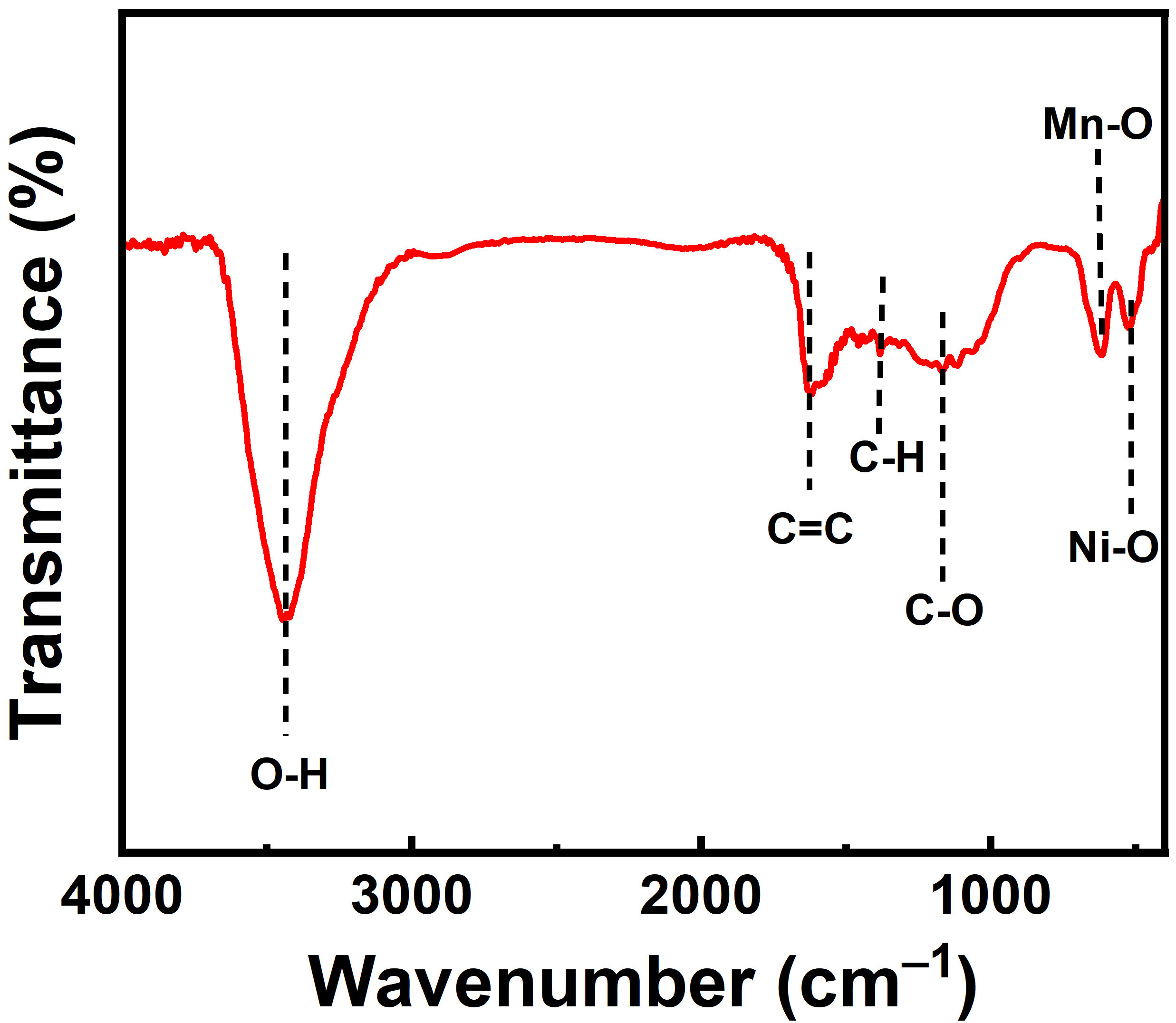

Supplement: Supplementary file 1 [file biosensors-13-00264-s001.zip › Revised Supplementary File/Figure S1.tif]

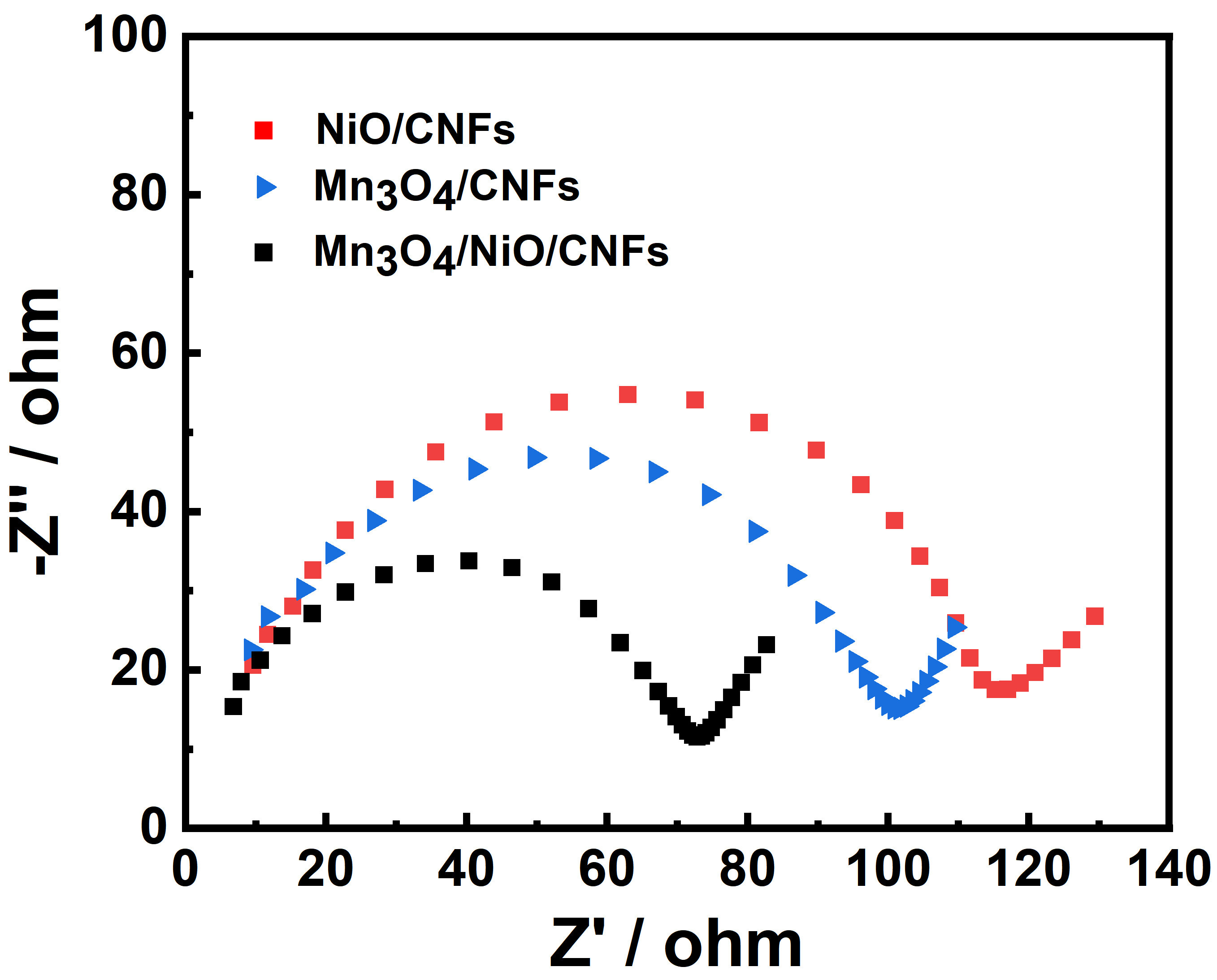

Supplement: Supplementary file 1 [file biosensors-13-00264-s001.zip › Revised Supplementary File/Figure S2.tif]

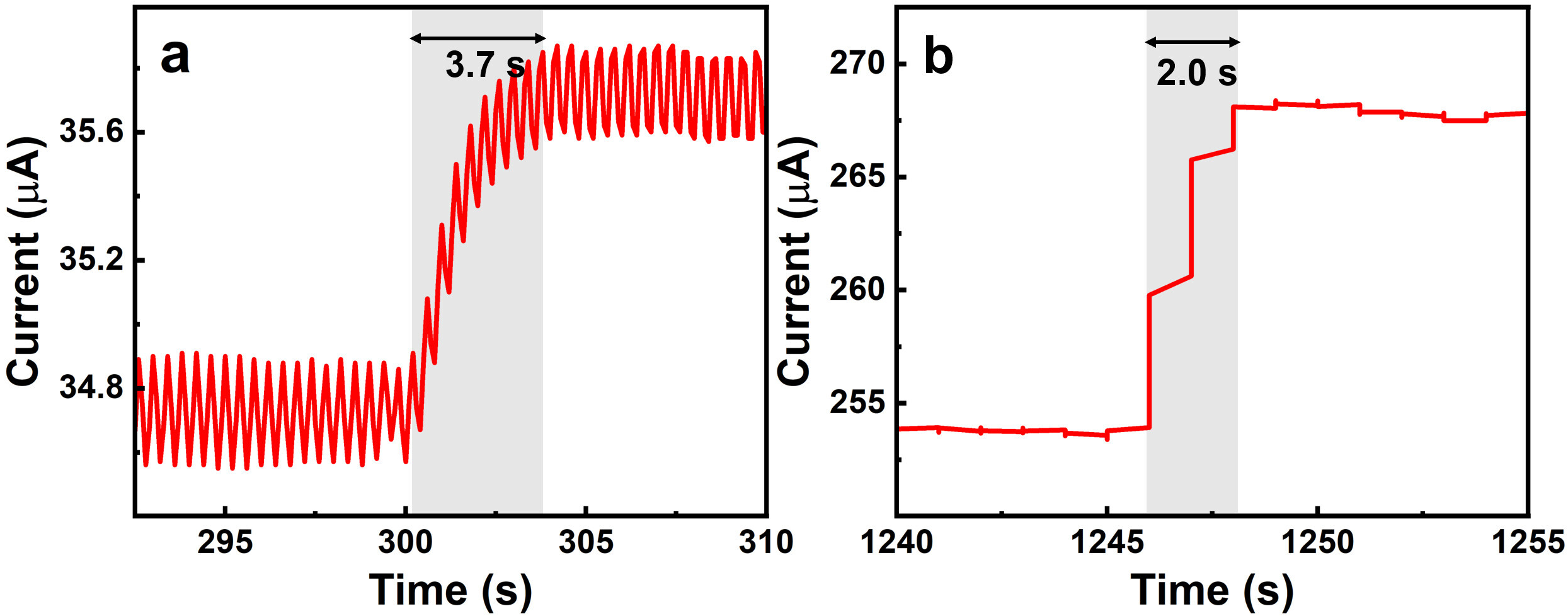

Supplement: Supplementary file 1 [file biosensors-13-00264-s001.zip › Revised Supplementary File/Figure S3.tif]

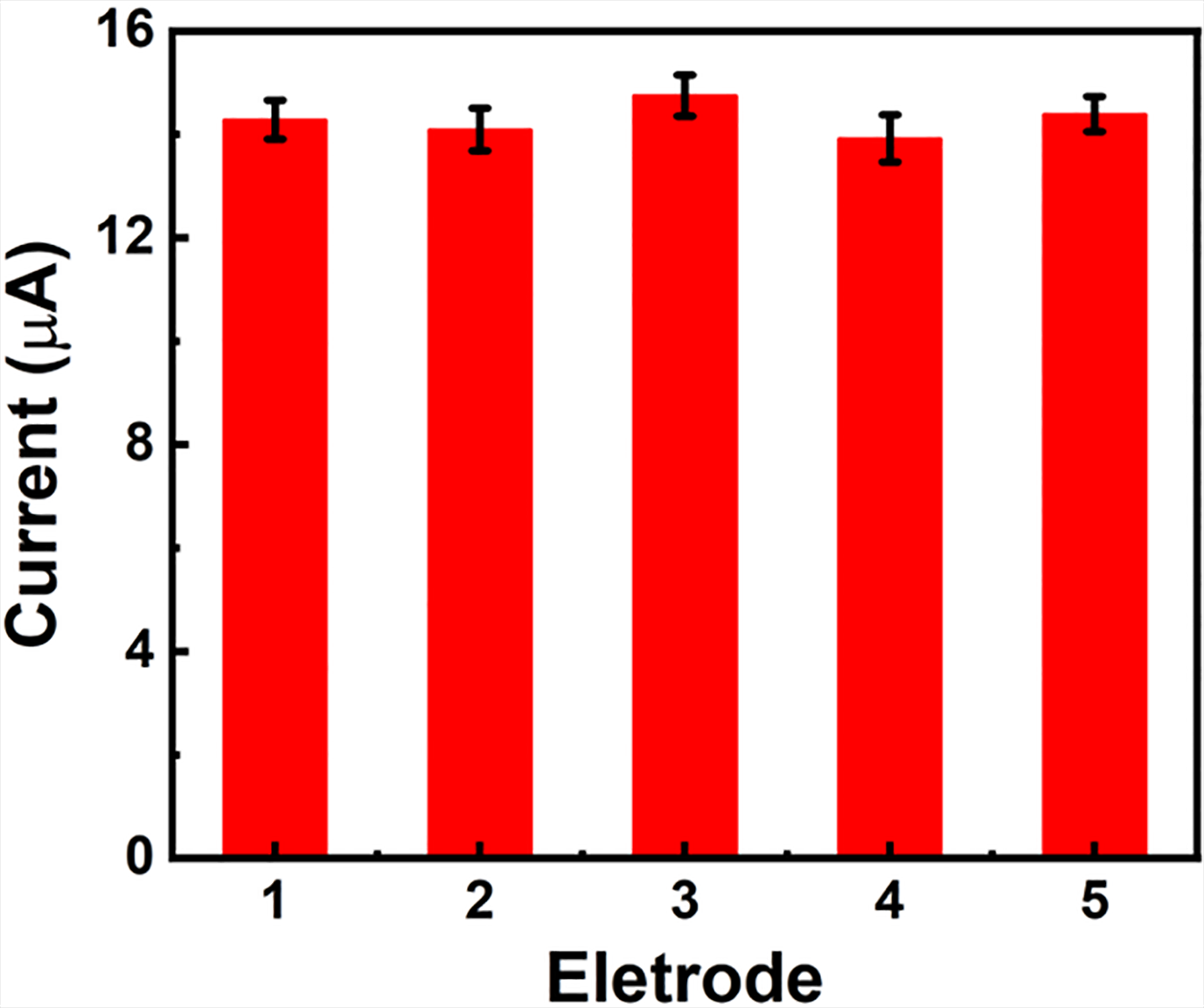

Supplement: Supplementary file 1 [file biosensors-13-00264-s001.zip › Revised Supplementary File/Figure S4.tif]

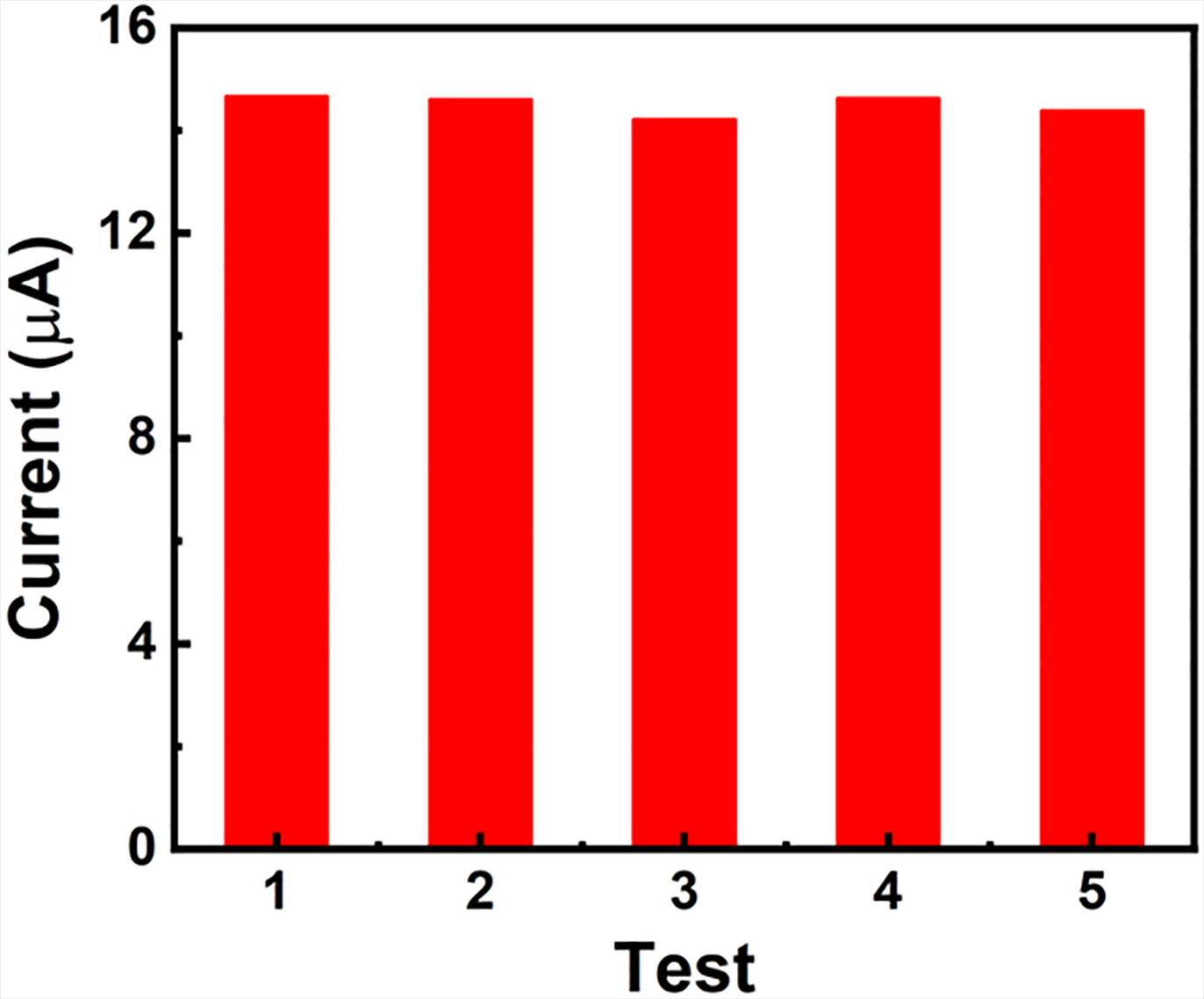

Supplement: Supplementary file 1 [file biosensors-13-00264-s001.zip › Revised Supplementary File/Figure S5.tif]

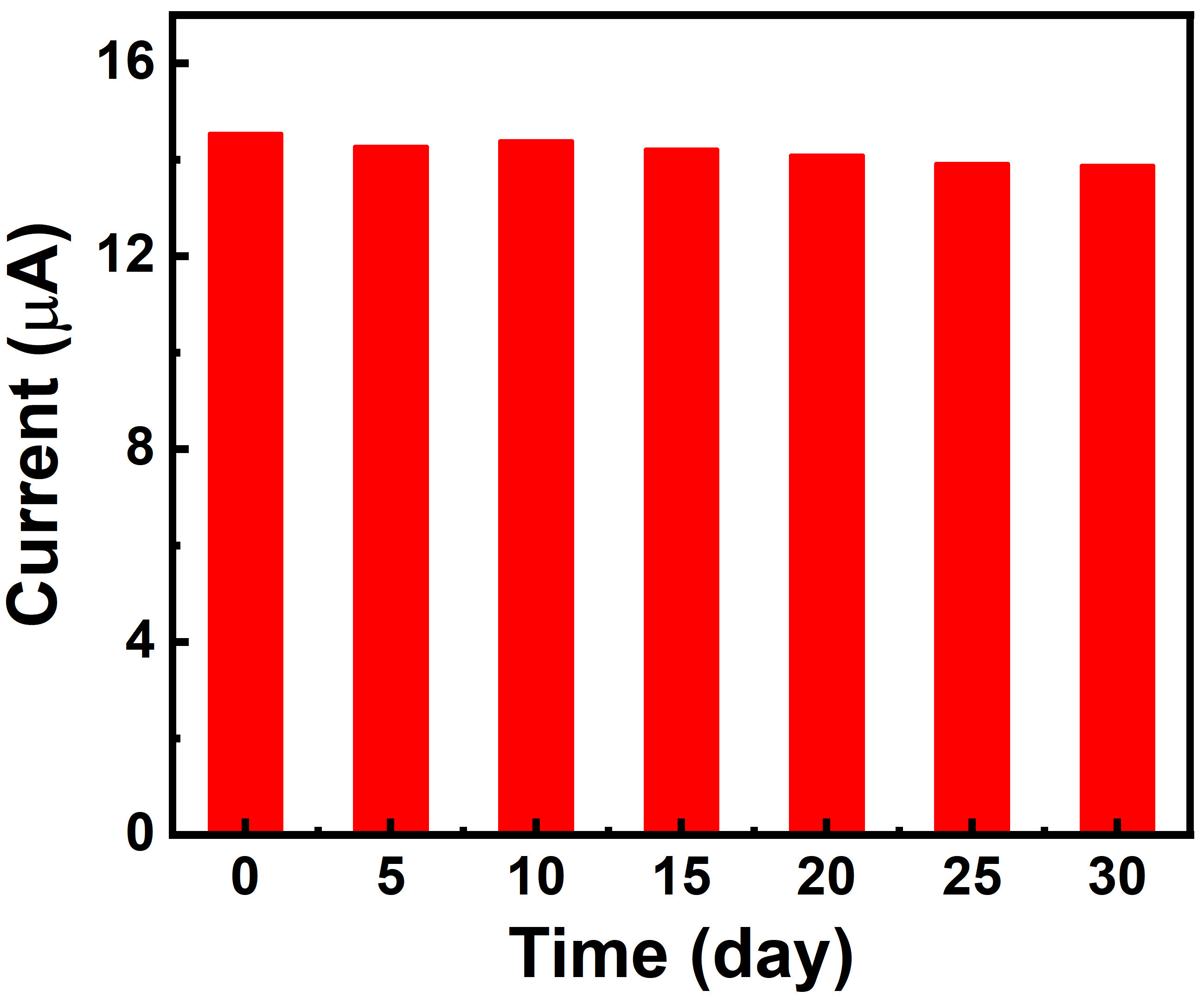

Supplement: Supplementary file 1 [file biosensors-13-00264-s001.zip › Revised Supplementary File/Figure S6.tif]
